# A surveillance of nosocomial candida infections: epidemiology and influences on mortalty in intensive care units

**DOI:** 10.11604/pamj.2014.19.398.4960

**Published:** 2014-12-22

**Authors:** Zehra Karacaer, Oral Oncul, Vedat Turhan, Levent Gorenek, Mustafa Ozyurt

**Affiliations:** 1 Etimesgut Military Hospital, Department of Infectious Diseases and Clinical Microbiology, Ankara, Turkey; 2 Gulhane Military Medical Academy, Haydarpasa Training Hospital, Department of Infectious Diseases and Clinical Microbiology, Istanbul, Turkey

**Keywords:** Nosocomiyal infection, candida, surveillance, mortality

## Abstract

**Introduction:**

It was aimed to investigate the frequency of Candida infections (CI) in the intensive care units (ICU), to determine typing of candida to evaluate risk factors associated with CI and mortality, and to evaluate influence of CI on mortality.

**Methods:**

The prospective cohort study was carried out between Jan 1, 2009 and Dec 31, 2010 in ICUs, and the patients were observed with active surveillance. VITEK 2 Compact System (BioMerieux, France) kits were used for the identification of isolates from various clinical samples.

**Results:**

A total of 2362 patients had enrolled for 16135 patients-days into the study. During the study, 63 (27,5%) of patients developed 77 episodes of CI were observed. Of the patients; 54% were male, 46% were female. Duration of hospitalization (OR = 1,03, p = 0,007), hyperglycemia (OR = 17,93, p = 0,009), and co-infections (OR = 3,98, p = 0,001) were identified as independent risk factors for CI. The most common infections were bloodstream (53%). 77 of 135 candida strains was isolated as causative pathogens. C. albicans (63,6%) was the most frequent species. Overall mortality rate was 78%. The rates of mortality attributable to CI and candidemia were 27%, and 18,3% respectively. Species- specific mortality rates of C.albicans and C.tropicalis were determined as 12%. High APACHE II scores (OR = 1,37; p = 0,002), and the use of central venous catheter (OR = 9,01; p = 0,049) were assigned as independent risk factors for mortality.

**Conclusion:**

CI is an important problem in our hospital. CI and associated mortality can be prevented by controlling of risk factors. Updating of epidemiological data is required for successful antifungal treatment.

## Introduction

Technological advances of today introduce more effective diagnosis and treatment methods to the medical world. However, these advances lead to the development of a population prone to infection as a result of new and powerful treatments and increasing medical applications administered along with primary diseases. Hospital infection (HI) incidence increases in this patients group. HI, being one of the most significant health problems, affects minimum five out of 100 patients followed up by hospitalization in USA, and one fourth of all HI is determined in intensive care units (ICU) [1, 2]. Candida infections (CI), most frequently encountered fungal infection (FI) among HI, are observed in a rate of 11% according to 2006-2007 CDC data [3].

FI has a large agent range and can be found in various clinical forms in immunosuppressive patients. However, symptoms of any one of them do not display characteristics distinctive from bacterial infections. Colonization and infection could be confused since many of them can be found in flora as well. It is not possible to make some diagnostic tests due to general state of the patients. Therefore, diagnosis is made by the analysis of known risk factors and clinical findings. Risk factors facilitating CI development are listed as central venous catheter (CVC) usage, broad-spectrum antibiotics usage, surgical intervention (specifically destruction of gastrointestinal system wall integrity), diabetes mellitus, renal failure, hemodialysis, total parental nutrition (TPN), immunosuppressive treatment, cancer and chemotherapy, transplantation, long term hospitalization and stay in ICU, colonization in different areas, and high APACHE II score (>20) [3]. It is clear that these factors point to patients whose general status is poor. The picture gets worse when CI is added in this group of patients.

In an evaluation discussing changing epidemiology of invasive fungal illnesses in Europe, it is indicated that mortality due to candidemia changes between 28-59% according to the population, species and geographical regions. In these infections with rather poor diagnosis, risk factors related to mortality are known as aging, seriousness of underlying illnesses, persisting infection, allogenic bone marrow transplantation, septic shock, and not being use antifungal prophylaxis application [4]. In this patient group with high mortality, definitive diagnosis is not always possible and preemptive and empirical treatment approaches are applied and antifungal agents are selected appropriate to the species prevalent in that center and according to the antifungal sensitivity pattern [5–7]. A decrease in candidemia frequency was determined in empirical treatment in febril neutropenic patients [8]. However, success of these approaches depends on the currency of epidemiological data of that center.

Distribution of pathogens frequently observed in patient groups and antifungal sensitivity rates may differ within years because of changing in treatment and patient groups. Failures to be caused by this situation can be prevented by close follow up and good analysis of culture results and periodically performed antifungal sensitivity tests. More patients started to be followed up in ICU in our center with the increase of patient transfer from various institutions by new arrangements. Problematic infections gained currency as a result of the transportation of hematology, oncology and thoracic medicine services with the service buildings in our hospital and with the addition of the flora of our immunosuppressive patients. HI incidence rate was estimated to be 12.4/1000 patient hospitalization days in 2007 when the start of the said change started in our hospital [9]. However, according to the surveillance data in the following year, this rate increased to 32/1000 patient hospitalization and CI rate was estimated to be 12.4%. Owing to the determination of growing nosocomial CI problem in our center, an obligation arisen for the tackling of matters that were not evaluated previously such as epidemiological data renewal, mortality ratios, and risk factors. This two-year surveillance study was planned for achieving high treatment success in our patients.

## Methods

**Study design**: our investigation was conducted in five ICUs (anesthesia, general surgery, neurology, internal medicine, and neurosurgical) and burn service of a teaching hospital with 1000 beds prospectively and in a controlled manner during January 1, 2009 - December 31, 2010. Agent isolation and diagnosis during the study period was done in the laboratories of Department of Infectious Diseases and Clinical Microbiology and Department of Microbiology and Clinical Microbiology.

**Ethical consideration**: Military Medical Academy Ethics Committee for Research in Health approval was obtained and permission to carry out the study was obtained.

**Criteria of inclusion/exclusion from the study**: criteria of inpatients, developing HI or healthcare-associated infections, Candida spp isolation, and evaluation of the isolates as infectious agents were sought without considering differences of age, gender and ICU in patients included in the study. The patients were evaluated in terms of infection findings according to the criteria of Disease Control and Prevention (CDC). Culture results of patients who do not have the diagnosis criteria were interpreted as contamination or colonization. Repeating attacks were followed up. In the event that infection of a new area or re-infection was determined, the patient's isolate was evaluated again as a study strain. In case the isolate was diagnosed as Candida spp. as a result of microbiological investigation, it was included in the study for further examination. Patients who do not appropriate HI definition were excluded from the study. These patients were followed up until discharge and included in the study if they met the criteria. Their proper treatments were planned when an infection was in question by other pathogens. Agents other than Candida were not included.

**Characteristics of the cohort**: our study was planned to be prospective and controlled. Age, gender, clinic of follow up, reason for primary hospitalization, applied surgical treatments, comorbidity, APACHE II score were attempted to be matched. It was paid attention for the control patients to be followed up in ICU minimum three days, use of at least one invasive tool, and minimum seven days of treatment by at least two wide-spectrum antibiotics. Patient group and cohort group were compared in terms of mortality development and risk factors.

**Patient follow-up**: hospitalized patients were followed up daily by the active surveillance method. Demographic characteristics, risk factors associated with candidiasis, developed infections, infection agents, clinical applications and their results were recorded. Changes in the clinical signs and symptoms of the patients and their laboratory results were evaluated in terms of HI development. The data obtained were interpreted daily by using CDC criteria updated in 2008. Required culture samples were taken from cases suspected to develop HI and subsequently, according to the current antibiotic sensitivity profile of our hospital, empiric or pre-empiric treatment protocol was started for the possible agent. Antifungal treatment of patients was rearranged following the diagnosis of the fungus. Treatment was applied to the patients as long as the period suggested by the current guides and they continued to be followed up until discharge in terms of new infection development.

**Microorganism diagnosis and stocking**: culture samples were cultivated in general and selective medium. The cultures were followed up for a period of 24-72 hours and the reproductions were evaluated. Isolation from blood samples were taken by BACTEC blood culture system. Microscopic evaluation was made from samples taken from the blood culture bottles in the tool giving reproduction signal, and subcultures were made from the ones displaFIng fungi cells in medium of Saboroud Dextrose Agar (SDA). Results of the microscopic studies made on other cultures displaFIng reproductions were evaluated similarly. In Candida identification, VITEK 2 compact system (BiomMerieux, France) was used to benefit from their biochemical characteristics. All of the isolates were purified by a single colony culture and kept in beaded storage tubes including bouillon with 15% glycerin at -800C.

**Evaluation of mortality results**: in a study investigating mortality, the following criteria were applied to decide mortality that is attributed to Candidemia [10]: 1) positive blood culture for Candida spp. within 48 hours before death; 2) persistent fever, hypotension and positive culture from clinically involved sites, suggesting persistent Candidiasis; 3) presence of disseminated Candidiasis in the autopsy; 4) relating the patient death strongly with Candidiasis by the clinicians. Based on this study, it was decided for the relationship of Candida with mortality by using other criteria, because autopsy was not able to be performed in our patients.

Mortality attributed to CI was estimated by formula 1 [11], and mortality specific to Candida species was estimated by formula 2 [10].

Mortality rate attributed to CI = Number of CI-associated mortality /number of patients with CI x 100 (Formula 1).

Mortality rate attributed to Candida species = Number of mortality caused by that species/Number of mortality in patients with CI x 100 (Formula 2).

Risk factors associated with mortality were similar to risk factors questioned for CI. Significant risk factors and independent risk factors were determined by similar statistical methods.

**Statistical Analysis**: all of the data received in the study was analyzed by collecting them in a SPSS 15.0 packet program. Categorical and measurable frequency distribution was analyzed by Chi-square (X2) and Mann Whitney-U analysis. Dependent risk factors were determined by single-variable analysis method, and independent risk factors were determined by multi-variable logistic regression analysis. P value of less than 0.05 was accepted significant.

## Results

**Surveillance results and demographic characteristics**: in our ICUs, 2362 patients were followed up by active surveillance for a total of 16135 hospitalization days, and 331 HI was detected in 229 patients. HI rate was estimated to be 14%, and the incidence rate of HI incidence density was estimated to be 20,5 in 1000-patients day. In the study, 77 CI was determined in 63 patients (27,5%). CIs make up 23,2% of all HIs observed in this period. The majority of patients were male (54%). Median age was 70,2 years and median APACHE II scores were 24,6. Women/men ratio of the patients making up the control group was 29/34, age average was 69,6, average APACHE II scores were 24, and there was no significant difference determined with the CI group (p= 0,79, p= 1, p= 0,66). However, hospitalization and ICU stay duration in CI group were found to be significant in comparison to the other group (p = 0,001, p = 0,01) (Table 1). When they were compared in terms of chronic illnesses, 14% diabetes mellitus and renal failure were observed in CI-developing cases and these ratios were found to be 27% and 22% respectively in the cohort. When the risk factors were compared in terms of CI development, antibiotic use, TPN use, mechanical ventilator (MV) use and chronic illness were found higher in the patient group, yet there was no statistically significant difference was determined. Hospitalization duration (p = 0,001), ICU stay period (p = 0,01), CVC use (p = 0,04), hyperglycemia (p = 0,001) and co-infections (p = 0) were determined to be risk factors for CI development. Among these factors, hospitalization duration (OR = 1,03, p = 0,007) and hyperglycemia (OR = 17,93, p = 0,009), co-infections (OR = 3,98, p = 0,001) were the independent risk factors (Table 1, Table 2). CIs determined in ICUs are made of 41 (53%) bloodstream infection (BSI), 22 (28%) urinary stract infection (UTI), eight (10%) pneumonia, four (5%) catheter associated-bloodstream infection (CABSI), and two (3%) soft tissue infection (STI).

**Table 1:** Demographic characteristics of the patients and cohorts and surveillance data

	Patients			Cohorts			P
	N	%		N	%		
Gender							
Female	29	46		29	46		1
Male	34	54		34	54		
Result							
Alive patients	14	22,2		31	49,2		0,002*
Mortality	49	77,7		32	50,8		
	Mean	St.D	Min-Max	Mean	St.D	Min-Max	
Age	70,22	19,48	14-95	69,59	19,56	14-95	0,79
Hospital day	46,78	36,73	5-190	26,08	16,15	0-80	0,001*
Hospital day in ICU	32,87	36,86	0-190	17,17	13,39	0-60	0,01*
APACHE II score	24,65	9,59	4-50	24	9,42	4-50	0,66
CVC day	17,83	28,24	0-129	8,43	11,76	0-46	0,05
UC day	22,75	28,36	0-129	17,06	12,92	0-60	0,99
MV day	12,63	25,53	0-121	6,17	10,48	0-46	0,3
Antibiotic# day	15,08	13,76	0-67	14,92	9,05	6-47	0,31

CVC: Central venous catheter, UC: Urinary catheter, ICU: Intensive care unit, MR: Mechanical ventilation

* Statistically significant

# The duration of antibiotic therapy are used before positive culture in patients, the duration of antibiotic therapy are used during follow-up period in cohorts.

**Table 2:** Risk factors associated with candida infection, results of univariate analysis and multivariate analysis

Risk factors	Patient			Cohort		P
	N	%		N	%	
Antibiotic therapy	61	96,8		60	95,2	0,64
Antacid therapy	56	88,9		60	95,2	0,18
UC	54	85,7		56	88,9	0,5
TPN	48	76,2		45	71,4	0,54
Chronic disease	46	73		42	66,7	0,43
CVC	44	69,8		33	52,4	0,04*
MR	35	55,6		26	41,3	0,1
Hyperglycemia	12	19		1	1,6	0,001*
Co- infections	45	71,4		21	33,3	0*
Risk factors	OR	95 CI	P			
Hospital day	1,03	1,01-1,05	0,007			
Hyperglycemia	17,93	2,03-158,49	0,009			
Co- infections	3,98	1,72-9,21	0,001			

CVC: Central venous catheter, UC: Urinary catheter, TPN: total parenteral nutrition

* Statistically significant

A total of 282 Candida species were isolated out of patient samples arriving to Microbiology and Clinical Microbiology Service laboratory in our Hospital, and it was determined that 135 of them belonged to the patients followed up in ICUs. As a result of the evaluation, it was determined that 77 Candida isolates isolated from 63 patients were HI pathogen. Out of the isolated Candida species, 49 (64%) were C. albicans, 11 (14%) were C. tropicalis, 5 (6%) were C. glabrata, 5 (6%) were C.parapsilosis, 3 (4%) were C. kefyr, 3 (4%) were C. dubliniensis and 1 (1%) was C. zeylanoides species. When the distribution of the infection agents according to the frequently seen CI was studied, 61% [25] of BSI was C. albicans and the others were (6; 14,6%) C. tropicalis, (4; 9,7%) C. glabrata, (4; 9,7%) C.parapsilosis, (1; 2,4%) C.dubliniensis, (1; 2,4%) C.zeylanoides originated. UTI pathogens were C.albicans (13; 59%), C.tropicalis (3; 13,6), C.kefyr (3; 13,6%), C.dubliniensis (2; 9%), and C.parapsilosis (1; 4,5%), respectively. It was determined that causative agents of pneumonia were C. albicans (5; 62,5%), C. tropicalis (2; 25%), C. glabrata (1;%12,5). C. albicans was determined on all STI and CRBSI.

**Mortality results**: during our study, 49 (78%) of the patients who developed CI and 32 (51%) of the patients in the cohorts died, and significance difference was determined in the two groups in terms of mortality development (p = 0,002) (Table 1). 26 (53%) of the patients with CI who died were women, 23 (47%) were men, and the median age of the patients was 75, and the median APACHE II scores was 28. In the comparison of the patients with CI who died with the patients with CI who survived, it was determined that there was significant difference in terms of age (p = 0), APACHE II scores (p = 0), and ICU stay duration (p = 0,02) (Table 3). In the patients who were diagnosed with CI and observed mortality, significant risk factors were determined for mortality in terms of gender (p = 0,04), age (p = 0), stay duration in ICU (p = 0,02), APACHE II scores (p = 0), antibiotic usage (p = 0,007), chronic illness (p = 0,004), azotaemia (p = 0,03), CVC use (p = 0,01), UC use (p = 0,001), and MV use (p = 0). Among these factors, APACHE II scores (OR = 1,37, P = 0,002) and CVC (OR = 9,01; p = 0,049) use were independent risk factors (Table 3, Table 4). Mortality associated with Candida spp. was determined in 17 patients who have CI and 12 were women, 5 were men. 9 of the mortalities depended on BSI, 4 depended on UTI and 4 depended on pneumonia. Responsible pathogens, on the other hand, were six C.albicans and C.tropicalis, two C.glabrata and Page C.dubliniensis, one C.kefyr.

**Table 3:** Demographic characteristics of the patients who were developed mortality attributed to candida infection and surveillance data

	Mortality			Survival			*P* value
	N	%		N	%		
Gender							
Female	26	53,1		3	21,4		0,04*
Male	23	46,9		11	78,6		
	Mean	St.D	Min-Max	Mean	St.D	Min-Max	
Age	75	12,39	37-95	53,5	29,23	14-89	0*
APACHE II Scores	27,61	8,02	10-50	14,2	7,22	4-30	0*
Hospital day	45,43	37,67	5-190	51,5	34,1	10-130	0,59
Hospital day in ICU	38,61	39,28	0-190	12,8	14,89	0-47	0,02*
CVC day	20,02	29,57	0-129	10,14	22,19	0-84	0,25
UC day	26,41	30,09	0-129	9,9	16,21	0-52	0,05
MV day	15,27	27,97	0-121	3,43	10,02	0-37	0,12
Antibiotic# day	16,53	14,51	1-67	10	9,46	0-30	0,11

CVC: Central venous catheter, UC: Urinary catheter, ICU: Intensive care unit, MR: Mechanical ventilation

* Statistically significant

# The duration of antibiotic therapy are used before positive culture in patients, the duration of antibiotic therapy are used during follow-up period in cohorts

**Table 4:** Risk factors associated with mortality among patients developed candida infection results of univariate analysis and multivariate analysis

Risk factors	Mortality			Survival		*P*
	N	%		N	%	
Antibiotic therapy	61	96,8		60	95,2	0,007*
UC	54	85,7		56	88,9	0,001*
TPN	48	76,2		45	71,4	0,05
Chronic disease	46	73		42	66,7	0,004*
Co-infections	45	71,4		21	33,3	0,18
CVC	44	69,8		33	52,4	0,01*
MV	35	55,6		26	41,3	0*
Azotaemia	24	38,1		18	28,6	0,03*
Risk factors	OR	95 CI	*P*			
		
APACHE II Scores	1,37	1,38-1,13	0,002			
CVC	9,01	1,01-80,18	0,049			

CVC: Central venous catheter, UC: Urinary catheter, TPN: total parenteral nutrition, MV: Mechanical ventilation

* Statistically significant

Accordingly, mortality rate attributed to CI was 27%, mortality rate attributed to infection was 18,3 in BSI, 8,1% in UTI, 8,1% in pneumonia, and species specific mortality rates were 12,2% in C. albicans, 12,2% in C. tropicalis, 4% in C. glabrata, 4% in C. dubliniensis, and 2% in C. kefyr.

## Discussion

Our study is a prospective and controlled investigation determining the place of CIs within HIs during a two-year period in a tertiary medical center with 1000-bed capacity. HI incidence was estimated to be 20,5/ 1000 patient-days and HI rate was 14% during the study period in our Hospital, and 12,4% of HI causes was Candida in 2007, and it elevated to 23,2% during the past two years. There is no evaluation made previously revealing the gradually increasing CI frequency in our ICUs, risk factors, mortality rates and risk factors that affect mortality. Discussion of this group of infections whose diagnosis and treatment is difficult will provide necessary information for the solution of this problem in other centers with similar characteristics. HIs, which are a general problem, were found as 26%, 19,8%, and 4,3% in ICUs in some studies conducted in this country [2, 12, 13]. CIs make up a significant section of HIs. In an investigation conducted in ICUs located in USA and Brazil, invasive Candidiasis was found to be 3%, and CI was determined to be between 8,5-10,3% in various studies conducted during 2000-2008 in this country [1, 12–14]. HI rate in our ICUs was lower than that in many other centers. However, there was a gradual increase observed based on previous surveillance data in our center. Higher values of our CI rate during the recent period than those in other centers and our old data were alarming. It is evaluated that this fast change could be due to an increase in the number of patients followed up in our Hospital as a result of new arrangements at the end of 2007 and transferred from other centers, and in addition, due to inclusion of oncology, hematology, and thoracic medicine clinics in our central hospital during this period. Differences from other centers could be originated from the different medical applications in the patient population, follow up and treatment.

It is vital that risk factors are followed up for preventing CI development, and for possible diagnosis and timely application of correct treatments. Bustamante [15] discussed a Candida originated epidemic in their center and indicated that intravenous catheters, long periods of hospitalization, steroids, TPN, hyperglycemia and ICU stay were the risk factors. Pappas et.al [10] determined in their prospective observational study that any intravascular catheter use was in 99%, antibiotic use was 99%, TPN was 58%, UC use was 40%, MV use was 31%, and diabetes mellitus was 30%. It was observed that the most frequently determined risk factors in our patients who developed CI were similar to other studies. Stay period in the hospital and ICU, CVC use, hyperglycemia and co-infections were determined to be significant risk factors. Hospitalization duration, hyperglycemia and co-infections among these were independent risk factors. It is well known that CI frequency increased after 10 days [16, 17]. Tzar et al [18] determined hospitalization for more than 10 days in 75,5% of the patients who developed Candidemia, and ICU stay was determined in 30,6%. In our study, CIs were observed in a rate of 27,3% during the first 10 days, and they were developed in a 48% ratio during 11-30 days, and when the entire hospitalization period was concerned they were developed in a 72,7% ratio after 10 days. This situation supported the determination of hospitalization period as an independent risk factor. It is known that hyperglycemia diminishes immunity and increases tendency to many infections. Furthermore, hospitalization frequency and follow up periods are more in this patient group due to diabetic complications [19]. It was indicated that diabetes mellitus was among predisposing factors in a 6% ratio in a study, and in another study it was stated that over 120 mg/dl serum blood sugar increases Candidemia development 2,6 times [15, 20]. In our study, on the other hand, it was noteworthy that although diabetes mellitus was encountered in 14% of our patients who developed CI, and in 27% in the cohort, hyperglycemia was determined 12 times more in CI group patients. It is revealed that this situation increased CI development approximately 18 times in statistical evaluation. It was inferred in our study that hyperglycemia was more influential than the presence of diabetes mellitus.

More antibiotic use by the patients because of co-infections eases CIs. However, in our hospital, there was wide-spectrum antibiotic use for longer periods with prophylactic purpose in patients who were administered surgical treatment specifically for 3-7 days or because of abdomen perforation. Therefore, there was antibiotic use in a similar frequency and duration in the control group as well without the presence of infection, and statistically significant difference in terms of this was not able to be manifested. Presence of another infection necessitates antibiotic use and longer hospitalization and longer invasive procedure application. Even if the patients who developed CI were similar with the control group in terms of age, gender, APACHE II scores and followed up clinics, their stay in the hospital and ICUs and catheter use durations were longer than those of the control group. Thus, it can be acknowledged that several of the risk factors encountered frequently in our study were a result of the presence of other infections. In this respect, statistically significant difference between CI and control group was not stunning. The most frequently observed infections among CIs is UTI, BSI and pneumonia respectively. In a study conducted in this country, UTI was determined to be 66,6%, BSIs was 18,5%, and pneumonia was 11,1% among CIs, and in another study these ratios were found to be 59,3%, 15% and 13,3% respectively [12, 21]. It was determined in a study carried out in our hospital in 2007 that infections were distributed as 57,7% BSI, 32,7% UTI, and 7,7% pneumonia [22]. The most frequently infections in our study were 53% BSI, 28% UTI, and 10% pneumonia. BSI was determined more frequently, UTI and pneumonia were determined less frequently in our study in comparison to the data of other centers. However, distribution of CIs did not change considerably in comparison to the data of the previous years in our center. All Candida isolated from the urinary system and respiration system are not accepted as reasons based on the daily evaluation data of the Hospital Infection Control Committee (HICC), and the patients who do not have UTI and pneumonia diagnosis criteria are accepted as colonized. Positive blood culture is revealed by techniques which are common in many centers and reproductions in blood culture are interpreted as Candidemia almost always. When it was considered that similar results were obtained in comparison to the previous data of our hospital, it was thought that there may be an approach difference between the centers in UTI and pneumonia diagnosis therefore BSIs were determined more frequently in our hospital.

The most frequent cause of invasive CI is C. albicans. However, increase in azole group antifungal prophylaxis and invasive application lead to an increase in non albicans species, in neutropenic patients primarily C. glabrata and C. krusei [17, 23]. In a study on immunosuppressive patients in this country, C. albicans was determined in a rate of 44,3%, and in another study, it was determined in a rate of 52% [24, 25]. In an investigation carried out abroad on ICU patients who are not neutropenic, C. albicans frequency was determined to be 40%, and it was 52,4% in this country [18, 21]. As differences between albicans and non-albicans group, distribution within non-albicans species changes according to the different studies. In an investigation, C. tropicalis was isolated in a rate of 7,7%, C. glabrata and C. parapsilosis was isolated in a rate of 5,9% [26]. In another study, again C. tropicalis was in the first rank with 11,2% among non-albicans Candida species isolated in ICUs, and C. parapsilosis was the second with 10,2% and C. glabrata was the third with 5,8% [27]. Although C. albicans was the most frequent isolate again with 45,5% in another study in our hospital three years ago, non-albicans isolate differed as C. parapsilosis 22%, C. tropicalis 18,2%, and C. glabrata 13%. It was considered that a small C. parapsilosis epidemic experienced in this period could cause this [22]. In our study, C. albicans was the most frequently determined species with 64% rate, and C. tropicalis, C.glabrata, C.parapsilosis, C.kefyr, C.dubliniensis, and C.zeylanoides followed this. Failure to do, organ transplantation, and allogeneic hematopoietic stem cell transplantation (HSCT) requiring long term immune- suppression in our hospital, is a reason differentiating our study from other studies which indicate C. glabrata as a second causative agents. When the infection agents were evaluated according to the infections, C. albicans was first, and C. tropicalis was second among Candidemia agents in various studies in this country [23, 25]. In some studies in this country, on the other hand, C. parapsilosis was detected as the second frequent pathogen [28, 29]. Pappas et.al [10] and Aliyu et al [20] state that the most frequent BSI agent was C. albicans, and C. glabrata was the second causative agents. Our results frequency was as C.albicans > C.tropicalis > C.glabrata = C.parapsilosis. These varying results can be explained by difference in patient population between the centers. The most frequent cause of UTI and pneumonia was C. albicans in our study, as similar to other studies [17, 21, 22, 25]. Although the prognosis of CIs changes according to the center, the flora of hospital and patient group, it is rather poor. In a study of cancer patients, it was observed that 39% of the patients died and the highest mortality was seen in C. glabrata infections, and the rates in ICU patients in France scaled up to 60% [4]. In another review report, it was indicated that mortality rates attributed to invasive Candidasia were between 10-49% [30]. In a three-year research carried out in a local hospital in USA, it was determined that mortality due to Candidemia was determined to be between 34-52% [15]. According to a resource published in this country, mortality was observed due to Candidemia in a rate of 14,5%, and due to invasive Candidiasis in a rate of 40-50% [16]. In our study, mortality due to all causes in patients who developed CI was 78%, and mortality attributed to CI was estimated to be 27%. It was observed that our mortality rate was lower than that of many other centers.

Follow up of risk factors in addition to proper and timely treatment will also contribute to the prevention of mortality. In a study conducted in USA, rate of risk factors determined for mortality and their statistical meaning were arranged as >18 APACHE II scores 62,1%, p< 0,001, malignity 50,1%, p = 0,002, UC use was 49,2% p = 0,004, arterial catheter use was 55,1% p< 0,001, corticosteroid use was 56,3% p< 0,001, being men 45,6% p< 0,004 [10]. It was determined in England that mortality increased with age (p = 0,0039), and complications associated with CI let to increased mortality of 6,5 times [20]. In our study, gender (p = 0,04), age (p = 0), ICU stay duration (p = 0,02), APACHE II scores (p = 0), antibiotic use (p= 0,007), chronic illness (p = 0,004), azotemia (p = 0,03), CVC use (p= 0,01), UC use (p = 0,001), and MV use (p = 0) were determined to be significant risk factors in terms of mortality, and APACHE II scores (OR = 1,37; p = 0,002) and CVC use (OR = 9,01; p = 0,049) were determined to be independent risk factors. Risk factors similar to other studies were revealed. It was determined distinctively that being women augmented mortality 4,14 fold (p = 0,04). In our patients displaFIng mortality, high APACHE II scores and CVC use rate was found to be 55,8% in women and 40% in men. The reason for the gender difference in terms of mortality could be the distribution of independent risk factors. When it was evaluated along with other risk factors, it was observed that all of them indicates unstable patients. The struggle with illnesses that have serious complications and difficult treatment like CI could become impossible in this group of patients. In our study, cause-specific mortality rates each Candida species were found to be 12,2% in C.albicans, 12,2% in C.tropicalis, 4% in C.glabrata, 4% in C.dubliniensis, and 2% in C.kefyr. In Pappas et.al study, these ratios were found to be 14% in C.albicans, and 12% in C.tropicalis, and 6% in C.glabrata and they were close to our findings [10]. Mortality rates associated with CI were found to be 18% in BSI and 8% in UTI and pneumonia. Although mortality depending on Candidemia varies according to publications, it was indicated to be between 14,5% and 59% [4, 16]. It was gratifying that results of our study remained under the data of some centers. There was no study found to evaluate mortality related to UTIs and pneumonia. Determination of risk factors associated with mortality to be between 50-100% in our patients who were UTI and died, and presence of MV support in 75% of our patients with pneumonia and presence of other risk factors can explain mortality.

## Conclusion

Although CIs augment in our hospital, non-albicans species are determined less often. However, with the change of patient profile in time, Candida spp. distribution may change as to affect their prognosis and treatment protocols. Similarly, mortality rates attributed to CIs may rise in time as a result of increasing resistant strains. It is possible to control some risk factors which affect the development of CIs and mortality. Rigorous application of infection control measures and follow up of epidemiologic data which change by means of similar studies should cause high treatment success in CIs.
